# Oscillators and servomechanisms in orientation and navigation, and sometimes in cognition

**DOI:** 10.1098/rspb.2022.0237

**Published:** 2022-05-11

**Authors:** Ken Cheng

**Affiliations:** School of Natural Sciences, Macquarie University, Sydney, North Ryde, NSW 2109, Australia

**Keywords:** bacteria, *Paramecium*, *Caenorhabditis elegans*, fly larvae, ant, sea turtle

## Abstract

Navigational mechanisms have been characterized as servomechanisms. A navigational servomechanism specifies a goal state to strive for. Discrepancies between the perceived current state and the goal state specify error. Servomechanisms adjust the course of travel to reduce the error. I now add that navigational servomechanisms work with oscillators, periodic movements of effectors that drive locomotion. I illustrate this concept selectively over a vast range of scales of travel from micrometres in bacteria to thousands of kilometres in sea turtles. The servomechanisms differ in sophistication, with some interrupting forward motion occasionally or changing travel speed in kineses and others adjusting the direction of travel in taxes. I suggest that in other realms of life as well, especially in cognition, servomechanisms work with oscillators.

## 1. Introduction

A classic concept views orientation in animals in servomechanistic terms [1]. This notion has been extended to navigation in animals [2,3]. Different criteria for goals to strive for set the standard for different navigational servomechanisms to aim for. Comparing current sensory or perceptual information with the goal state delivers an error signal based on the discrepancy between the current state and the goal state. In servomechanisms, errors drive movement to reduce the error. When the error is minimized, the animal should be heading in the correct direction or else at the goal.

For example, in path integration [4,5], the animal keeps track of the distance and direction from its starting point, typically its home. When it is time to return home, the servomechanism works to reduce the calculated vector to zero. In view-based navigation, a navigating ant might be judging the familiarity of the scene [6] or be driven by both attractive views and repellent views (which are to be avoided) [7]. Another model has a pair of servomechanisms driving the ant to turn left when the best view is to the left and to turn right when the best view is to the right [8].

My focus on servomechanisms had concentrated on the representational bases that form the goals for these goal-directed systems to aim for [2,3]. While I still think in terms of representations of goals for servomechanisms, this focus has neglected how the actions of organisms fulfil such goals [9]. In orientation and navigation, I now add that oscillatory processes [10], invoking another classic notion [11] (von Holst in [12], ch. 5), usually fulfil this role. Servomechanisms work with oscillators in various ways, to be spelled out shortly. An oscillator produces orderly endogenously generated action on a periodic basis (Box 1). The oscillator and the servomechanism have both been called basic units of action (along with the reflex [12]). The movements of effectors that move organisms, from legs to fins to cilia, typically operate as oscillating systems [10]. Gallistel defined oscillators in neural terms as being generated by a neural pacemaker unit that pulses periodically [12]. Oscillations, however, are rife in non-neural organisms, such as single-celled eukaryotes and prokaryotes [16,22], which move with regular beats of cilia and flagella, and even in physical phenomena, such as the rotations of planets around a sun. For my purpose here, I consider any form of periodic movement of effectors as oscillators, regardless of whether such movements are generated neurally or not.

Box 1. **How are orderly oscillations generated?**

The mechanisms differ in neural and non-neural organisms. Neural organisms use neurons to coordinate oscillations while non-neural organisms must rely on other means of coordination. In some cases, the mechanisms are still unknown. This box sketches selected cases.

Neurally endowed animals rely on pacemakers to generate oscillations [12,14]. Pacemakers are single neurons or groups of neurons that fire on a regular basis. With appropriate connections to downstream effectors, periodic action is generated. The key to effective action, especially in locomotion, is to coordinate different oscillators, such as those driving limbs in insects [12,14] or muscles on the dorsal and ventral sides of the nematode *C. elegans* [15].

In the slime mould *Physarum polycephalum*, a multi-nucleated, single-celled amoeboid, oscillations are generated in the fluid in the cytoplasm by regular contractions of muscle-like actomyosin fibres of the cell wall [16,17]. In ciliated organisms such as *Paramecium*, an army of cilia on the outside beat in coordinated waves. The bases of coordination is still unclear. Half a century ago, the thinking was that a combination of external factors such as hydrodynamic flow and internal physiological factors make for coordinated beating [13,18]. Half a century later, although the modelling is more sophisticated, the story line contains the same two classes of external and internal factors, perhaps differing across organisms [19]. In *Escherichia coli* and *Salmonella enterica*, movement is generated by the beating of flagella. When a motor turns counterclockwise, all the flagella bundle up to make one long tail, and the oscillatory beating of the tail moves the organism forward [20,21].

In all these oscillatory systems, the oscillations are modifiable, subject to internal and external factors. They do not run willy nilly without control. The description of how oscillations are modified for orientation and navigation forms the core of this paper.

The concept of servomechanisms in concert with oscillators applies to both orientation and navigation, processes that I distinguish. In orientation, the organism attempts to reach a better place by some criteria (e.g. higher concentration of food), but not any particular place. A fruit fly larva or *Paramecium* travelling up a chemical gradient of food provides an example. In navigation, the organism attempts to reach one particular place. A homing ant returning to the one nest that is its own provides an example.

I illustrate this modified servomechanistic concept with a non-exhaustive number of examples. Then I discuss why this mode of operation found in orientation and navigation also pervades other domains in life, especially in cognition: in perception, attention and working memory.

## 2. Servomechanisms and oscillators in concert: cases

**Figure 1.**
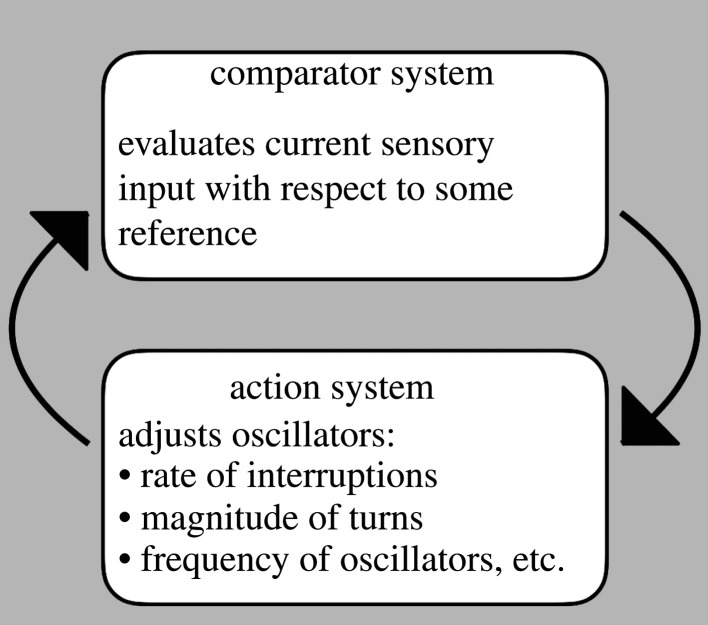
The overarching conceptual scheme. A representational system, the comparator system, compares current input with some reference, which might be whether a chemical gradient has gone up or down or the familiarity of a panoramic view. This comparison process leads to adjustments to oscillators with different degrees of intimacy between the servomechanism and the oscillators. At the minimal end, forward movement is stopped occasionally by interrupting the oscillations that drive locomotion. In this case, it does not matter what is driving the movement; it happens that most locomotion is powered by oscillations. At the most intimate end, the magnitudes of turns in oscillations are adjusted based on sensory feedback. In such cases, having oscillations is crucial for the servomechanism to work. The adjustments and actions in turn feed back by way of changed sensory input to complete a loop, which is what makes the system a servomechanism.

To set the stage for the cooperative play between servomechanisms and oscillators, which span a vast range in scale from tiny bacteria travelling micrometres to giant sea turtles roaming thousands of kilometres, different cases differ in the intimacy of cooperation between the two players (figure 1). At the least intimate end, the servomechanism interrupts ongoing locomotion occasionally, with the rate of interruptions being adjusted according to input conditions. Oscillators happen to be used by most organisms of all sizes to locomote. At the intimate end, transverse or left-right oscillations are adjusted depending on feedback. Here, the oscillations provide essential material for the servomechanism to operate on: if the animals do not move with transverse oscillations—the cases that I could find are all animals—the servomechanism cannot function. In an intermediate case, that of sea turtles, the oscillations powering locomotion are tweaked to keep on course. I will not attempt an exhaustive review here but wish to illustrate these different motifs as well as the enormous range.

**Figure 2.**
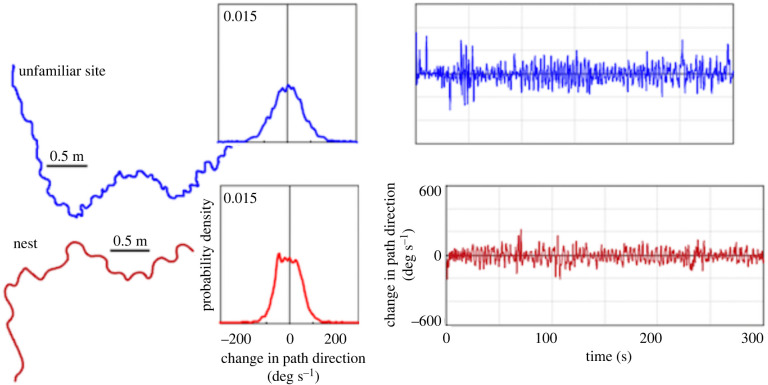
Two examples of transverse oscillations in bull ants, *Myrmecia croslandi*. The ants were placed on a styrofoam ball (trackball) floating on air, supporting their own weight while they walked, and the actions were filmed from above. The trackball was either at an unfamiliar site (top row) or else at the ant's nest (bottom row). The left panels show paths as calculated from movements on the trackball. The middle panels show the distribution of changes in heading direction. The right panels show changes in path direction over time. From [7], reprinted with permission. (Online version in colour.)

Starting with a well-studied case, ants exhibit all the motifs. These eusocial insects navigate with two layers of oscillatory movements. The six legs need to oscillate, to push off, lift, and plant, in coupled fashion to orchestrate the tripod gait, which is most often used in walking [12,14,23,24]. In a tripod gait, the front and rear legs of one side are coupled together with the middle leg of the opposite side. On top of these coupled oscillations, ants also oscillate left and right [7,8,25]. At larger amplitudes, these side-to-side oscillations are visible when observed by eye [26]; at a finer scale, they can be documented with ants walking on a trackball that is floating on air [7] (figure 2). Adjustments to the tripod gait make for changes in speed of travel [23,24]. In one adjustment illustrating the intermediate motif of servomechanisms tweaking oscillators, as ants approach their nest, the speed of travel decreases systematically [27]. Adjustments to both suites of oscillations undergird navigation.

In navigation, ants modulate their transverse oscillations in servomechanistic fashion [8,10,25]. Basically, when the navigation is going well, oscillations are small in amplitude. With any form of uncertainty or unfamiliarity in the surrounding cues, however, the amplitude of what has been called ‘meandering’ [26] increases, and the ants slow down. Adjustments to transverse or side-to-side oscillations require adjustments to the tripod gait, a process that has not been described in detail yet. Illustrating the interruption motif, ants also occasionally interrupt their forward movement, stopping the side-to-side oscillations and the coupled leg oscillations, and turn their bodies in saccadic fashion to pause and look in various directions [28]. The rate of these scanning bouts that interrupt oscillations also increases when the navigation is not going well. For example, both meandering and scanning increase if an ant that has almost reached home is picked up by an experimenter and placed back somewhere on the route that it had just traversed, a manipulation called rewinding [29], or when an ant has fallen into a pit trap on the previous trip that took a while to escape from [30]. In bull ants (genus *Myrmecia*) as well, unfamiliar visual conditions induce scanning behaviour (*M. croslandi* [31]; *M. midas* [32]). Unlike other cases to come, however, these interruptions do not end with random re-orientation of travel direction. Both meandering and scanning are thought to supply the navigating ant with visual information that might inform it of a better route to travel.

Two smaller, much-studied animals also illustrate both the interruption motif and the intimate motif of cooperation between servomechanisms and oscillators: the nematode *Caenorhabditis elegans* [33,34] and the larvae of *Drosophila melanogaster* [35]. The small-brained *C. elegans*, with 302 neurons, practices a chemotaxic mechanism called weathervaning in which it gradually curves towards the peak of a chemical gradient [33,34]. Swings of the head—where chemosensors reside—left and right to compare chemical concentrations supply crucial information for the chemotaxis. Turns can then be biased towards the favourable side. In neural modelling based on an artificial-evolutionary algorithm, a four-element system can account for the chemotaxis. ON and OFF neurons, which react to increases and decreases in the ‘desired’ senory gradient, respectively, control the performance of motor neurons signalling the dorsal and ventral muscles [36]. The modelling shows that the OFF neuron, making adjustments to decreasing gradients, plays the more prominent role in orchestrating chemotaxis.

The term ‘taxis’ has been linked to the notion of a servomechanism [1,3]. In the more neurally endowed *Drosophila*, fly larvae orchestrate chemotaxis as a chief means of ascending or descending chemical gradients [35]. The larvae move with two kinds of oscillations, each requiring coupled oscillations of muscles. Peristaltic oscillations shortening and lengthening the body wriggle the larva forwards. Simultaneously, another form of oscillations (unrelated in timing) wiggles the larva from side to side much like ant locomotion in navigation. In the transverse oscillations, the better side draws bigger turns towards it. Thus, if things get better on the left, the larva turns left more than it turns right. The servomechanism biases the larva to travel up (or down) a gradient, and it ends up milling about the region of highest (or lowest) concentration. The brain of the fly larva, with fewer than 10 000 neurons [37], is not needed for oscillation-based movement but is required for chemotaxis [38].

Transverse oscillations carving out zigzag movements are also practiced by flying insects such as silkmoths, *Bombyx mori* [39]. The strategy increases the chances of coming across the sought-after sensory cue, in the case of male silkmoths, pheromone emitted by a conspecific female. The strategy is so common that Namiki and Kanzaki tabled 22 different species of arthropods that have shown this mode of travel [39]. In orienting to an odour source, various vertebrate animals, including fishes such as eels and salmon and a range of seabirds, also exhibit zigzag motion [40]. Salmon and eels display a variant of zigzag motion by oscillating vertically instead of horizontally.

As for the interruption motif, the roundworm *C. elegans*, along with non-neural organisms, the eukaryote *Paramecium* and the prokaryotes *Escherichia coli* and *Salmonella enterica*, all stop forward movement based on oscillations occasionally in orienting up or down chemical gradients. The basic pattern, known as chemokinesis (details in Box 2), may be embellished by refinements to improve orientation. Chemokinesis should be differentiated from chemotaxis, although the term ‘chemotaxis’ is often applied to cases of chemokinesis. In chemokinesis, the sensed chemical gradient changes the rate of certain behaviours (a change in rate featuring as the key characteristic of any kinesis), whereas in chemotaxis, the mechanism picks out a better direction of travel.

Box 2. **Movements in *E. coli*, *S. enterica*, *Paramecium* and *C. elegans***

As described in Box 1, in bacteria *E. coli* and *S. enterica*, a constantly turning motor in the counterclockwise direction produces coordinated flagellar beating. Occasionally, the motor turns in the opposite direction, the flagella come apart, and the bacterium takes a tumble, turning in a random direction (figure 4). *S. enterica* can bias its tumbles to orient in smaller turn angles by untwisting fewer than the full complement of flagella [21]. *Paramecium* moves by beating many cilia on the outside of its body in coordinated oscillations [41,42]. The eukaryote interrupts its movement occasionally by what is called an avoiding reaction, which is a behaviour that it executes should it bump into something [43]. The *Paramecium* springs back, turning in a random direction. A similar spring is performed by the nematode *C. elegans* [15,44], which moves by coordinated coupled oscillations of its muscles on the dorsal and ventral sides.

In *E. coli* [45] and *C. elegans* [46], the interruptions take place as a function of one (bacterium) or two (nematode) Poisson processes. Poisson processes are random-rate processes in which the chance of an event occurring at any point in time is constant. The result is many short inter-event intervals and an overall (negative) exponential distribution of inter-event intervals. The nature of inter-event intervals has not been examined in *Paramecium* as far as I know. As already discussed, ants also occasionally interrupt their movement to scan, both in navigation [10,28] and in initial learning walks around their nest before becoming a forager [47–49]. It would be theoretically important to examine inter-bout intervals to determine if a random-rate (Poisson) process governs these scanning bouts or whether a more regular (non-random) process is at play. Wood lice and *Paramecium* also change locomotion speed in another form of kinesis, orthokinesis. This can be accomplished by increasing or decreasing the frequency of oscillators. Fruit fly larvae modify the amplitude—another parameter of oscillatory systems—to turn kinesis into taxis in tracking chemical gradients [35].

In nematodes [15,34,44,46], *Paramecium* [15,43] and bacteria [15,20,21,45], their rates of occasional interruptions of forward movement fall under servomechanistic control. In these interruptions, the forward movement stops, and the organism turns in a quasi-random direction and then heads off in this new direction. The basic strategy is that if the gradient is getting better, the rate of interruptions decreases, while if the gradient is not getting better, the rate of interruptions increases. The result of this servomechanistic control is to bias the movement to end up in regions of better chemical concentrations. In the peak region, the going does not get better, and these organisms end up milling about there. Humans forced to adopt this form of servomechanism in a computer game also manage to orient to the designated goal area [50].

Various adornments boost the interruption motif. In *C. elegans*, turns are biased toward bigger turn angles [46], and the much-studied worm also appears to try different directions in rapid succession with interruptions of movement with turns (the interruption is called a pirouette [44], the same term that is applied to saccades of scans in ants [51]) until it finds an improving gradient, after which it tends to maintain a mostly straight course, called a run [34,52]. The nematodes pull off this seemingly purposeful search-until-you-find-the-way servomechanism because reactions to worsening gradients are quick while reactions to an improving gradient are slower; the two are based on different neural mechanisms featuring different neurons.

Even non-neural organisms embellish the basic interruption-based chemokinesis. After an interruption, the new direction appears to be completely random in *E. coli* [20,45] and *Paramecium* [43]. In the bacterium *S. enterica*, on the other hand, turns when the going gets worse are randomly distributed, while turns when the going gets better are biased towards smaller angles [21]. The bias comes about from fewer than the full complement of flagella being unbundled in the tumble.

*Paramecium* adds another kind of kinesis to its repertoire, orthokinesis [1,43]: speed of movement is modulated based on sensory gradients. *Paramecium* exhibits orthokinesis based on chemical cues [43], with the mechanism playing a significant role in orientation when the rate of turns is low. In repulsive conditions, they move faster, while in attractive conditions, they move slower. Orthokinesis is also practiced by the neurally endowed wood louse (*Porcellio scaber*) in seeking out moister regions [53]. The behaviour is called hygrokinesis (‘hygro’ having to do with humidity). They move faster in drier regions and slow down or become motionless in moister regions. The basic mantra in orthokineses goes: When the going is bad, go faster; when the going is getting better, slow down.

At the large-scale end and illustrating the third, intermediate motif, seven species of sea turtles roam the oceans of Earth for thousands of kilometres on their sojourns [10,54]. In the Atlantic Ocean, they need to keep within a safe zone in a giant region known as the Sargasso Sea. Turtles use primarily geomagnetic cues for orientation (electronic supplementary material, Box S1).

**Figure 3.**
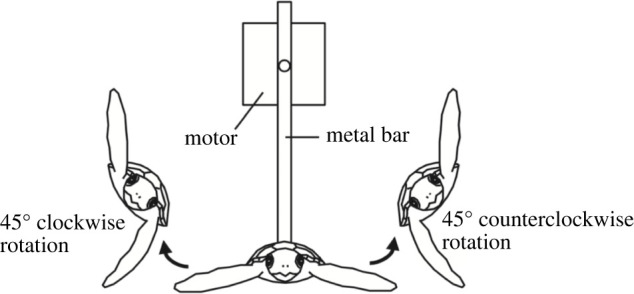
How sea turtles correct disturbances in roll. The experimental animal was strapped to a harness and lifted in air. It was experimentally rolled to make one side lower than the other. The turtle adjusts its power stroke so that one flipper is lower than the other while stroking. From [55], fig. 2, reprinted with permission.

Knowing which way to go, however, is not enough. The turtles also have to keep on track in a particular direction in the face of turbulent waves and wind that knock them off in all three dimensions of rotation, yaw, pitch and roll. Servomechanisms keep them on course by modifying their oscillating swimming motion [10,55]. Turtles swim by using power strokes of their front flippers oscillating in synchrony, as in a butterfly stroke [56]. This oscillating system is adjusted in the face of experimental displacements in yaw, pitch, or roll [55]. Against roll (rotations about the head-to-tail axis of the body), for example, the two flippers stroke at different angles (figure 3).

**Figure 4.**
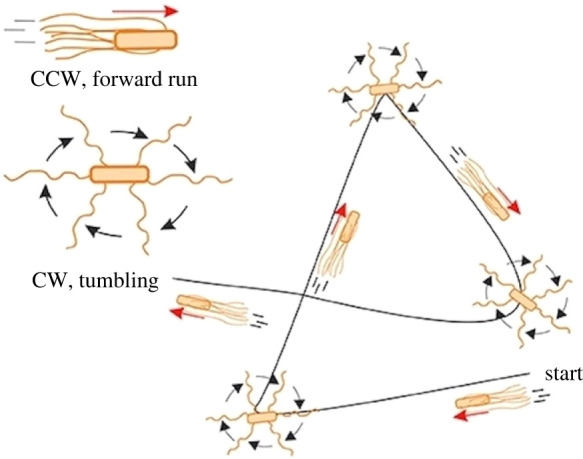
Run-and-tumble pattern of movement in bacteria (*E. coli*). When the motor driving the flagella turn in a counterclockwise (CCW) direction, the flagella bunch together and their beating drives the bacterium forwards. Occasionally, the motor reverses direction, the flagella come apart, and the organism tumbles to orient in a random direction. When the motor turns counterclockwise again, the prokaryote resumes forward movement in the new direction. From Wikimedia Creative Commons, https://commons.wikimedia.org/wiki/File:Swimming_strategy_of_bacteria_-_run_and_tumble.jpg. Authors: Julio Bastos-Arrieta, Ainhoa Revilla-Guarinos, William E. Uspal and Juliane Simmchen. Licence: https://creativecommons.org/licenses/by-sa/4.0/deed.en. (Online version in colour.)

These demonstrations of servomechanisms coping with disturbances [55] were conducted in artificial laboratory conditions different from the oceans in which the turtles travel. Unlike ants, turtles' movements and accompanying course control in their natural ocean habitat have not been detailed to my knowledge, despite ample sophisticated studies on turtles' orientation. It would be worth obtaining footage of turtles swimming in the ocean to conduct the kind of detailed analysis examined in ants. With drone technology now available, researchers could track turtles for at least the first small portion of their globe-spanning voyages.

## 3. Discussion

In orientation and navigation, from the micrometres of bacteria to the tens of thousands of kilometres of sea turtles, we find servomechanisms working with oscillators, the latter broadly construed. Different servomechanisms work with oscillators with different degrees of intimacy. The bacteria *E. coli* and *S. enterica*, the eukaryote *Paramecium* and the nematode *C. elegans* all interrupt the ongoing oscillator-based locomotion occasionally to re-orient the organism in a new direction, achieving kinesis. The link in these cases can be called incidental, in that the oscillators happen to be the devices driving the locomotion that is interrupted occasionally. This interruption-based mode is sometimes embellished with variations to make it more efficient. In *C. elegans*, *Drosophila* larvae and ants, another kind of servomechanism modifies properties of the oscillating system directly to carry out chemotaxis (worm, fly larvae) or navigation (ants). In these cases, transverse oscillatory movements furnish crucial ingredients for the servomechanisms to operate on. The link is intimate. With transverse oscillations, the use of a short memory of whether things are getting better or not results in taxes rather than kineses. In intermediate cases, the frequency of oscillators might be reduced to slow movement, as found in ants nearing their nest, or the way the oscillatory movements are carried out might be adjusted, as in sea turtles attempting to stay on course in a turbulent medium.

Transverse oscillations in ants—as opposed to oscillations that propel an organism straight forward such as peristalsis in fly larvae or flagellar beating in bacteria—allow frequent adjustments to head the traveller in the correct direction [8,57]. Side-to-side movements support course control even when facing the goal direction entails facing a non-distinctive uniform white surround [58]. The experimental space did contain a distinctive black landmark to the left of the target heading, but Woodgate *et al*. [58] painted the left eye of experimental wood ants so that they saw nothing but white when facing the target direction. Zigzagging allowed the ants to face the landmark direction frequently and then adjust their turns to travel in the target direction. Transverse oscillations have also been shown in modelling and robotics to aid course control in flying agents [59], although the extent to which this form of control operates in flying insects remains to be determined. In single cells, the zigzag movement has been hailed as a strategy to move farther in straight-line distance per unit time, as compared with random walks [60,61]. Thus, a range of locomotory functions can be found for transverse oscillations.

The nuances in working with oscillatory systems remind us how important it is to focus on actions and how they come to be carried out, a point made by Gallistel [12] over four decades ago, but sometimes forgotten in the cognitive revolution; I can include my own work as citations on this point [62–67] (see also [9]). Full understanding of mechanisms and functions of orientation and navigation can only come about with detailed attention to the actions of organisms beyond measures of headings of travel or places organisms arrive at.

Why is the theme of servomechanisms working with oscillators so common in orientation and navigation? One answer is surely that so many locomotory systems in mobile organisms rely on oscillators as defined here. For efficient locomotion, effectors need to move in coordinated fashion, and coupled oscillators coordinate effectors. This theme applies to the cilia and flagella of single-celled organisms [68,69], to the limbs of insects [12,14], or to the entire body of fish (von Holst in [12], ch. 4). Actions of any kind may also be inherently servomechanistic [70]. von Holst & Mittelstaedt [71] (in English in [12], ch. 7) formulated the idea of reafference. Copies of the efferent commands or reafference from sensory feedback form an important component of all actions. The motor system works with a comparator taking into account not only the effector output, but also the resulting reafferent pattern [70]. To oversimplify, the action system compares what is done with what is expected to be done. This notion applies to animals with brains. Whether it applies to animals without brains or to non-neural organisms remains an open question.

The realm of physiology also features some cases of servomechanistic control over oscillators. A key concept of physiology is homeostasis [72], a notion around which the concept of servomechanisms arose. Much of physiology concerns feedback systems to keep crucial variables within acceptable ranges for life. The mammalian heart illustrates servomechanistic control over an oscillating system. The heart contains three different pacemakers, two as back-ups for the main sino-atrial pacemaker [72]. The pacemaker coordinates muscle contractions in the heart to make a functional heartbeat. Intrinsic control within the heart and extrinsic control via input from the nervous system modulate the amplitude and frequency of heartbeats.

While bodily physiology sometimes relies on servomechanisms operating on oscillators, it is in cognition and the neurophysiology that undergirds it in which the theme has proliferated in the past decade. In neurophysiology, oscillations play major roles [70], with modulations of oscillations possibly providing key control over neural processes. This theme would require a large monograph to capture, but in primates, perception [73], attention [74], and working memory [73,75] all wax and wane in cycles in performance. If we consider such cognitive activities as goal-directed [76], then they can be considered servomechanisms relying on various neural oscillations for operation, a notion worth exploring theoretically and empirically. VanRullen [73] noted that although hints of such cycles surfaced in the 1960s, it requires sophisticated data gathering and, importantly, analytic techniques to sift the cyclic signals from the noise; VanRullen suggests that this explains why the theme did not flourish earlier.

Groups of neurons in mammalian brains beat in phase at different rhythms given Greek letters as names (alpha, beta, gamma, delta, theta, etc.). Phase relations may serve to gate the outputs of one group versus another for downstream receivers [77]. That is, if a sender group is connecting with a receiver group while the latter are in a receptive phase, as opposed to being in a refractory period, the sender's message gets through. Fries theorizes that this form of modulatory control over phase relations forms the gateway for attention.

Phase relations in one particular cycle, the theta wave, is said to modulate the output of hippocampal cells coding places, known as place cells [78]. Place cells typically fire when an animal is in a particular place in its environment [79]. Working on rodents, Sanders *et al.* [80,81] (see also [10]) posit that place cells firing in one half of the theta cycle code for the current place while place cells firing in the other half of the theta cycle code for future places where the rodent is heading to. The theta cycle may be gating information about external events from the hippocampus, forming a crucial component in navigational control.

It does not require a brain or a nervous system to use oscillations for servomechanisms, as the non-neural organisms in this review show. The slime mould *Physarum polycephalum*, called the ‘intelligent unicellular eukaryote’ ([82], p. 1; for reviews, see [83], p. 7; [84]), has fluids oscillating through its network of tubules in its large body. Working with these oscillations provides the means for *Physarum* to accomplish its many feats, a few of which are listed briefly here because a detailed explication would take a full paper to document. Tracking of gradients entails changes in local oscillators and the entrainment of other oscillators [85]. Adjustments to oscillations are implicated in learning [16], memory encoded in the tubule sizes within the body [82] and decision making [86]. A formal model of information transfer within the body of *Physarum* relies on adjusting oscillations [87]. Much of the cognition of this ‘intelligent’ slime mould fits our current theme, a notion that deserves a fuller exposition.

It is perhaps not surprising that servomechanisms and oscillators end up playing large roles in orientation and navigation—and perhaps much else in cognition. These units have been called basic units of action along with the reflex [12]. In this regard, even the reflex often has flavours of servomechanisms and oscillators. A large opus by Sherrington on reflex physiology (see [12], ch. 2) relied on the scratch reflex of dogs. Gallistel [12] pointed out at the start of his chapter on oscillators (ch. 4) that the scratch reflex showcases repeated scratching motions, very much oscillatory in nature. I can point out in addition that the rhythmic scratches are targeted at the spot where experimental stimulation was applied rather than anywhere else. The spectre of servomechanistic control over oscillators even pervades some reflexes. In sum, why not structure navigational routines out of basic units? Basic units would be evolutionary tidbits with which natural selection (the 'tinkerer' [88]) can fashion functional systems. Along with the lack of sophistication in handling data that VanRullen [73] pointed out, I think that a dominant focus on representations in the study of cognition of humans (in cognitive psychology) and other animals (comparative cognition) has clouded what might seem obvious from this review, that oscillations in action play a large role in cognition and indeed in life.

Looking ahead, I call for more focus on the actions of animals, beyond the concern with whether an animal can do X, as well as more focus on the behaviour of non-neural organisms. What an organism is doing in solving a problem or seeking a mate or food is of interest, and so is the distribution of behaviours in time. In problem-solving in animals, what cycles of attempts are found, perhaps reflecting cycles of motivation? In *Paramecium* and in ants, how are interruptions of locomotion distributed in time, in the case of ants, both in learning the visual environment initially [47–49] and in scanning at the start of a trip on a well-known route [28]? In the end, I think that endogenously generated oscillations (von Holst in [12]) feature as a common and effective strategy with which organisms organize their own activities, while servomechanisms constitute a major mode for adapting to the environment, including an organism's own internal environment—a broad theme for theoretical and empirical research. Together, these two basic units make the stuff of life.

## Data Availability

The electronic supplementary material is available online [89].
